# Ingar Olsen (1942–2024) – in memoriam

**DOI:** 10.1080/20002297.2025.2503944

**Published:** 2025-05-25

**Authors:** Bruce J. Paster

**Affiliations:** ADA-Forsyth Institute, Cambridge, MA, USA

Professor Ingar Olsen, Editor Emeritus of the *Journal of Oral Microbiology*, passed away at the end of February 2024. He began the *Journal* in 2009 and retired in 2023. He was extremely proud of ‘his’ Journal as it became extremely successful in just a few short years. He was an extraordinarily insightful scientist – a role model to all (Figure 1).

**Figure:**
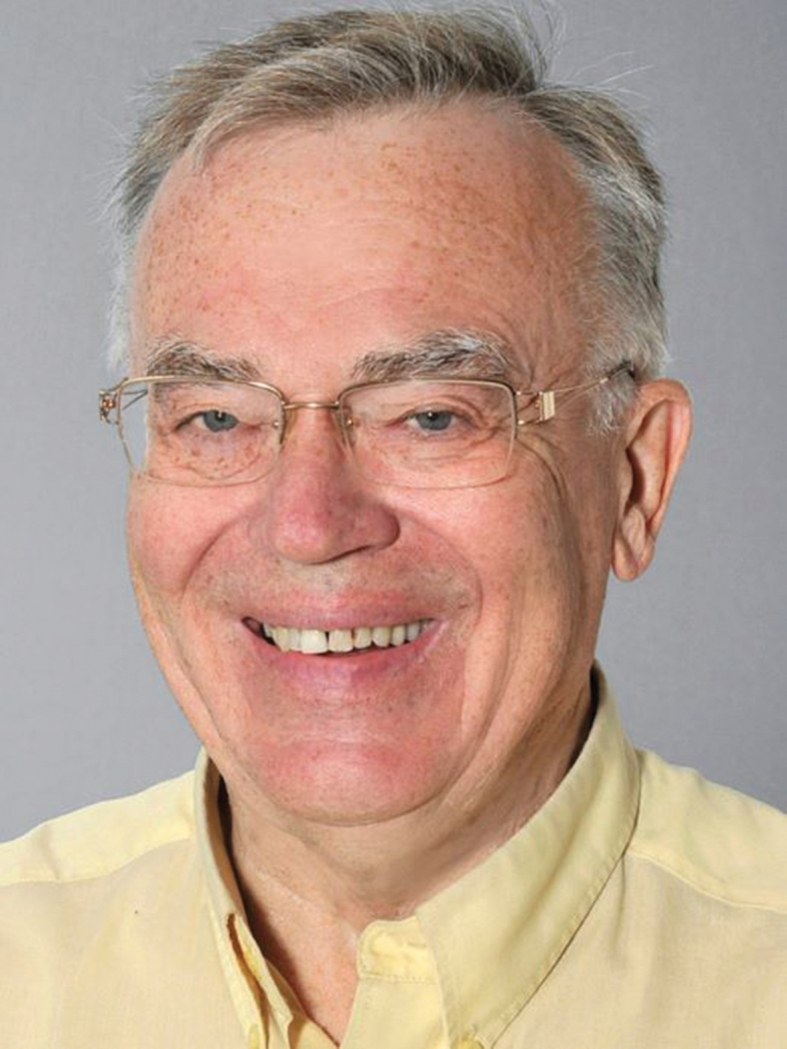

It is with great sadness to report that Professor Ingar Olsen, Editor Emeritus of the *Journal of Oral Microbiology*, passed away at the end of February 2024. He began the *Journal* in 2009 and retired in January 2023. He was Editor-in-Chief of the Journal.

Dr. Olsen received his dental degree from the Dental Faculty, University of Oslo, in 1966. He was atrainee in oral surgery in 1970, received his PhD in 1976, and in 1988, he became professor in microbiology at the Department of Oral Microbiology, University of Oslo. Dr Olsen was head of this department from 1987 to 1989. He was a visiting scientist many times to the Forsyth Institute (now ADA-Forsyth), to the Virginia Polytechnic Institute and State University and to Centraalbureau Voor Schimmelcultures in Delft Holland. Dr Olsen’s main research interest was chemotaxonomy of bacteria and yeasts and he co-authored several new bacterial species descriptions. In 2001, a new bacterial genus, *Olsenella* [1], was named in his honor. He is co-author of several textbook chapters in microbiology, editor of a textbook in dental hygiene and author/co-author of more than 200 scientific papers. Over the years, Dr Olsen supervised many master and PhD candidates in microbiology, periodontology, and endodontology. Another research passion of Dr. Olsen was anaerobic microbiology as it relates to periodontology and endodontology [2]. He was a master at culturing oral anaerobic bacterial species [1,3–5], especially those that were considered difficult to grow.

In 2009, Dr. Olsen had the foresight to begin a new online journal in the field of oral microbiology – the *Journal of Oral Microbiology*. At that time, he felt it was important to focus on the recent developments in molecular biology. He knew that the microflora of the oral cavity was as important as the microflora found in the rest of the body. In addition to bacterial associations with many oral diseases, he published many works that implicated oral species that were also associated with several systemic diseases [6] or afflictions, including cardiovascular diseases [7], colorectal cancer, pancreatic cancer [8], preterm birth, diabetes [9], and Alzheimer’s disease [10–12].

Dr. Olsen was immensely proud of his journal. As Editor-in-Chief, with his leadership and perseverance, the *Journal* became very successful with a high impact factor. In his last year as EIC in 2022, the *Journal* had a 5-year impact factor of 5.2 and a CiteScore of 8.8. Indeed, he told me his goal was to achieve an IF of more than 5. He himself published 47 manuscripts in the *Journal* during his tenure. He officially retired from the *Journal* in January 2023.

Dr. Olsen meant so much to many. Listed below are remembrance quotes responding to his passing – all had a common thread. He was an exceptionally insightful scientist, an inspiration to all, a colleague, and a dear friend. He was a kind and gentle person with an easy laugh.

Ingar … had a strong sense of esprit de corps among oral microbiologists and immunologists as a leader in oral health sciences … His passion for fundamental knowledge development and cross-cutting thinking in the field of oral and systemic health was inspiring. He was a paragon of modesty despite the major body of scientific work he produced and accomplishments he achieved, which made him a role model for younger scientists including me … Dr. Olsen served as a champion in empowering researchers to work together with clinicians for uncovering the significance of oral microbes that could play in general human health which reflected his ethos as a bona fide scientist and clinician.

Ӧzlem Yilmaz, Medical University of South Carolina

We were members of a grant consortium funded by the European Commission … He was an extremely kind person, and I will never forget him.

Jan Potempa, University of Louisville

He was indeed influential and an inspiration.

Mary Ellen Davey, ADA-Forsyth

I remember him as possibly the most kindest of us all. Always interested in new avenues, always interested in scientific discussion, and always prepared to share. I will remember Ingar as the perfect colleague.

*Arie Jan van Winkelhoff, LabOral, Netherlands*

I remember him for his kind and friendly presence at meetings and obviously his academic contribution to oral microbiology and the journal.

*William Wade, Kings College London*

His experimental work and work with his journal cannot be overestimated. It is impressive that he was able to establish such a valuable and respected scientific journal. *Mogens Killian*

I have known Ingar since he came to Socransky’s lab in the 1980’s and remember him as kind, gentle but insightful person.

*Anne Tanner, ADA-Forsyth*

I was familiar with his significant contributions to the field of oral microbiology and admired his work. His legacy, especially with the *Journal of Oral Microbiology*, will continue to inspire many in our community.

*Bat Bor, ADA-Forsyth*

Our most frequent and productive work took place at the International Taxonomic Subcommittee on Gram-Negative Anaerobic Non-Sporing Rods meetings which each of us chaired in succession. These meetings … were a hub of scientific and social exchange, where we frequently published reports which also led to us visiting each other’s laboratories. We held Ingar in great esteem, and the memories of our collaboration and wonderful times together will remain deeply cherished.

*Haroun N.* *Shah, Middlesex University & University of West London*

*Saheer E.* *Gharbia, Wellcome Sanger Genome Institute*

Thank you for passing along this “final page” of Ingar’s life. His career and Forsyth’s have many strong linkages as have been noted. He will be missed but his impact on oral micro remains strong.

*Dan Smith, ADA-Forsyth*

Professor Ingar Olsen´s genuine interest and wide research in microbiology for more than forty-five years gave him many friends and colleagues across the globe. He has always been a highly respected member of the scientific community, especially in oral microbiology, and the list of research papers are long … Ingar received a number of awards for his work, and we all will remember him for the kind and enthusiastic person he was, and a researcher with deep insight and knowledge and many achievements.

*Morten Enersen, Pål Brodin, Edward Messelt and colleagues and staff at the Faculty of Dentistry, Oslo, Norway*

Ingar was a great man, he will be greatly missed.
